# Attentional skills following traumatic brain injuries in elite female athletes

**DOI:** 10.1192/j.eurpsy.2025.2436

**Published:** 2025-08-26

**Authors:** N. Syrmos

**Affiliations:** 1 Aristotle University, Thessaloniki, Greece

## Abstract

**Introduction:**

Traumatic brain injuries are serious traumatic situations

**Objectives:**

Aim of this study was to evaluate the attentional skills in cases with traumatic brain injuries, in elite female athlete

**Methods:**

3 female elite athletes (1 basket ball player, 1 runner and 1 ping pong player ) were participated in this study, 60 days more or little less after cervical spine injuries. Range of age 25-35 years and mean age 29.

We used specific performance tests, letter cancellation exam, naming trials and iq tests.

**Results:**

All of them(3,100%) reported deficits in all areas of attention function and also cognitive and emotional status.2 of them(66%) reported also difficulty in complex task activities, especially when time limits were imposed.

**Conclusions:**

All these exams are good and efficient tools in order to evaluate this kind of patients.

**Disclosure of Interest:**

None Declared

